# Impact of Anthropogenic Activities on Microbial Community Structure in Riverbed Sediments of East Kazakhstan

**DOI:** 10.3390/microorganisms12020246

**Published:** 2024-01-24

**Authors:** Olga Muter, Dita Gudrā, Gulzhan Daumova, Zhanat Idrisheva, Marzhan Rakhymberdina, Guntis Tabors, Baiba Dirnēna, Linda Dobkeviča, Olga Petrova, Baitak Apshikur, Megija Luņģe, Dāvids Fridmanis, Igor Denissov, Yerkebulan Bekishev, Raimonds Kasparinskis, Zarina Mukulysova, Stanislav Polezhayev

**Affiliations:** 1 Faculty of Biology, University of Latvia, 1 Jelgavas Str., LV-1004 Riga, Latvia; guntis.tabors@lu.lv; 2 Latvian Biomedical Research and Study Centre, 1 Ratsupites Str., LV-1067 Riga, Latvia; dita.gudra@biomed.lu.lv (D.G.); megija.lunge@biomed.lu.lv (M.L.); davids@biomed.lu.lv (D.F.); 3 School of Geosciences, D. Serikbayev East Kazakhstan Technical University, 19, Serikbayev Str., Ust-Kamenogorsk 070000, Kazakhstan; gulzhan.daumova@mail.ru (G.D.); zhanat.idr@mail.ru (Z.I.); marzhanrakh@mail.ru (M.R.); opetv@mail.ru (O.P.); bake.ab@mail.ru (B.A.); idenisov@ektu.kz (I.D.); ybekishev@edu.ektu.kz (Y.B.); zarinab01@mail.ru (Z.M.); 4 Faculty of Geography and Earth Sciences, University of Latvia, 1 Jelgavas Str., LV-1004 Riga, Latvia; baiba.dirnena@lu.lv (B.D.); linda.dobkevica@lu.lv (L.D.); raimonds.kasparinskis@lu.lv (R.K.); 5 Center of Excellence “Veritas”, D. Serikbayev East Kazakhstan Technical University, 19, Serikbayev Str., Ust-Kamenogorsk 070000, Kazakhstan; staspolezhaev@mail.ru

**Keywords:** biodiversity, heavy metals, microbial community structure, multi-metal resistance, river sediments

## Abstract

Heavy metal (HMe) pollution in regions with mining and metallurgy activities is known to be a serious environmental problem worldwide. Hydrological processes contribute to the dissemination of HMes (drainage, precipitation, flow rate). The aim of the present study is to investigate the microbial community structure in ten river sediments sampled in different regions of East Kazakhstan, which are contaminated with HMes. The overall degree of sediment contamination with HMes (Cr, Cu, Zn, Pb, and Cd) was assessed using the pollution index Zc, which ranged from 0.43 to 21.6, with the highest in Ridder City (Zc = 21.6) and Ust-Kamenogorsk City, 0.8 km below the dam of the hydroelectric power station (Zc = 19.6). The tested samples considerably differed in organic matter, total carbon, nitrogen, and phosphorus content, as well as in the abundance of HMe-related functional gene families and antibiotic resistance genes. Metagenomic analysis of benthic microorganisms showed the prevalence of Proteobacteria (88.84–97.61%) and Actinobacteria (1.21–5.98%) at the phylum level in all samples. At the class level, Actinobacteria (21.68–57.48%), Betaproteobacteria (19.38–41.17%), and Alphaproteobacteria (10.0–39.78%) were the most common among the classified reads. To the best of our knowledge, this is the first study on the metagenomic characteristics of benthic microbial communities exposed to chronic HMe pressure in different regions of East Kazakhstan.

## 1. Introduction

Heavy metal (HMe) contamination is released into the environment through a variety of anthropogenic activitiremediation and resistance mechanism es, particularly mining and metallurgical processes, sewage discharge from plastic manufacturing, fertilizer production industries, and others [1]. This, in turn, poses a hazard to ecological systems and human health, affecting food supply chain stability and, hence, food security [2,3]. The dissemination of HMe through water flow, atmospheric deposition and sedimentation, snow cover, transboundary transfer, and other routes adversely affects soil, surface runoff, and infiltration [4]. As a result, short-term and chronic changes in the biodiversity of ecosystems occur. These changes are expected to be species- and site-specific, depending on geographical location, sediment geochemistry, climate conditions, concentration, combinations of HMes, contamination history, etc. In this respect, the shift in microbial community structures and functions in HMe-contaminated freshwater sediments represents an important area of research for several reasons.

First, HMes in sediments can affect important functions of microorganisms, e.g., respiration (because of the inhibition of the electron transport chain), cell division, nitrification, denitrification, functions related to the carbon cycle, and genetic information processes [3,5,6,7]. Hence, the inhibition of microbial communities by HMes can affect the biogeochemical cycling of nutrients.

Second, historically contaminated sites can serve as a source of “beneficial” microorganisms capable of HMe bioremediation via bioleaching, biosorption, biotransformation, biomineralization, and intracellular accumulation [8]. Bacterial isolates derived from HMe-contaminated sites can be further applied in a broad range of bioremediation technologies. Numerous studies have recently shown the potential benefits of isolates derived from HMe-contaminated sites. Thus, bacterial communities belonging to the genera *Shinella*, *Microbacterium*, *Micrococcus*, and *Bacillus* have been shown to have bioremediation potential related to HMes (Cd, Cr, Co, Ni, Zn) [9]. The biosorption of Cu by *Bacillus* spp. was shown by Danial and Dardir (2023) [10], while chromate reduction caused by *Arthrobacter* sp. SUK 1201 was reported by Dey and Paul (2016) [11]. Special attention can be paid to phytoremediation, which is based on plant–microbe interactions caused by rhizofiltration, phytostabilization, phytoextraction, phytostabilization, and phytovolatilization [5,12]. Furthermore, bacteria belonging to Actinobacteria, Firmicutes, and Proteobacteria help fish *Pterygoplichthys pardalis* overcome HMe stress in their intestines [13].

Third, HMe-tolerant bacteria can be cross-resistant toward organic pollutants; e.g., HMe-tolerant *Bacillus cereus* BCS1 degrades pyrethroid [5], and furthermore, HMe tolerance among bacterial species derived from hydrocarbon-contaminated sites has been detected [14]. Arsenic-resistant bacteria have been isolated from uranium ore [9]. In addition, some microorganisms are genetically resistant to HMes and can regulate metal bioavailability in the environment [15]. All these phenomena could considerably facilitate bioremediation processes in multi-compound-contaminated sites. On the other hand, HMes can increase the level of antibiotic resistance. Specifically, the occurrence of antibiotic resistance co-selection is typical in the sites contaminated with HMes [16]. However, the effects of HMes on freshwater sediment microorganisms and the mechanisms of microbial resistance toward HMes are poorly understood.

Our study focused on a comparative analysis of microbial community structures in HMe-contaminated river sediments in the East Kazakhstan region. One of the main sectors of the economy of the Republic of Kazakhstan is the mining and metallurgical industry. Previously published studies conducted in East Kazakhstan region reported high levels of contamination with Cr, Zn, Pb, and Cu in soil, groundwater, and surface waters near metallurgical enterprises [17,18]. Increased HMe content in meltwater has also been detected in urban areas near metallurgical enterprises, which subsequently enters the soil, groundwater, and surface water, polluting them [19]. The Irtysh River flows through the territories of three countries: China (618 km), Kazakhstan (1589 km), and Russia (2041 km) [20]. The accumulation of soluble HMes in the Irtysh River near large cities leads to the formation of complex geochemical anomalies. Chen and Wang recently studied the impact of urbanization on the functional traits of macroinvertebrates within the Irtysh River Basin, comparing samples at 17 sites. A gradual transition was detected, with an increase in pollution-tolerant taxa. The urban region was characterized by the strongest niche occupation, resource utilization, and buffering capacity for environmental fluctuations [21].

Despite intensive studies on geochemical and ecological changes caused by HMe pressure, data on the responses of microorganisms to HMe contamination in river basins in the East Kazakhstan region are scarce. The present study is aimed at investigating the microbial community structure in sediments of Irtysh, Ulba, Tikhaya, and Krasnoyarka Rivers in Ust-Kamenogorsk City, Ridder City, and Glubokoye District, dependent on the distance of sampling sites from metallurgical enterprises. It is hypothesized that microbial communities inhabiting historically contaminated river sediments represent unique compositions of multi-metal-resistant taxa. Determining the HMe concentrations and physicochemical characteristics of river sediments, on the one hand, and metagenomic analysis of benthic microorganisms, on the other hand, will provide new knowledge related to microbial ecology in extreme environments, particularly chronic HMe stress. Bacterial communities play critical roles in biogeochemical cycles and serve as sensitive indicators of environmental fluctuation.

## 2. Materials and Methods

### 2.1. River Sediment Sample Collection and Processing

**Figure 1:**
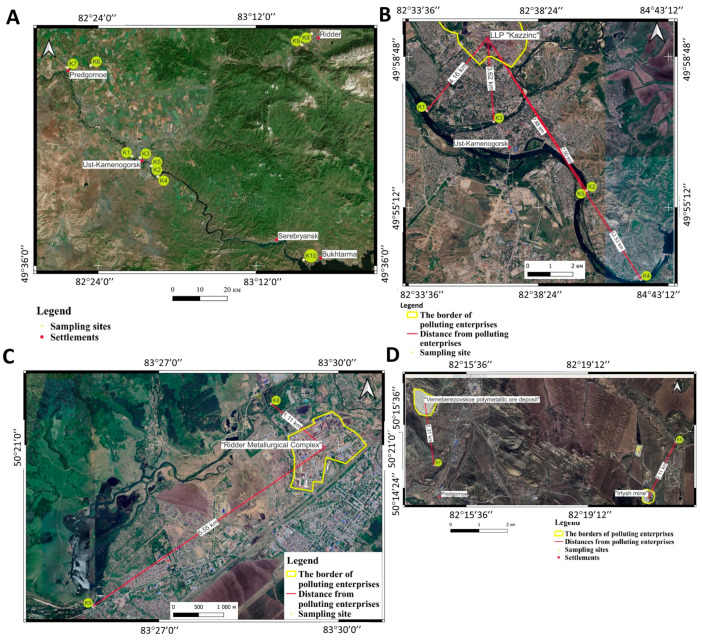
Location of sampling sites: (**A**) area of ten sampling sites; (**B**) Ust-Kamenogorsk City; (**C**) Ridder City; (**D**) Glubokoye District.

River sediments from ten sites were collected in October 2023. The locations of the sampling sites are shown in Figure 1 and described in Table 1. Sampling sites for bottom sediments are concentrated near industrial facilities. Figure 1B shows the distances of the sampling sites from the large metallurgical company Kazzinc LLP, located within the city of Ust-Kamenogorsk. In the city of Ridder, the main facility that has a negative impact on the environment is the Ridersky metallurgical complex (Figure 1C). In the Glubokoye District, a significant share of environmental pollution comes from the Irtysh Copper Smelter (IMZ), which operated from 1937 to 2002 and is currently closed. Ore for processing came from the Verneberezovskoye deposit to the Belousovsky processing plant, which includes the Irtysh mine (Figure 1D). To construct the maps, Bing Satellite images with a spatial resolution of 0.5 m per pixel were used. Data processing and map construction were carried out in the QGIS program. OpenStreetMap was used as a base map to obtain toponymic information (names of rivers, villages).

Sampling was carried out using a stainless-steel bottom grab with a capture area of at least 0.025 m^2^. The part of the collected sample (500 g) that did not touch the walls of the sampler was separated and placed in a previously prepared sterile plastic container for sample transporting and storage. In total, 10 point samples of bottom sediments were taken. The collected samples were immediately delivered to the lab and divided into three parts, i.e., for metal determination (stored at 4 °C for 24 h), chemical testing, and metagenome analysis (stored at −20 °C). The selected sediment samples were delivered to the laboratory of the Veritas Center of Excellence (EKTU), where preliminary sample preparation was carried out for laboratory research in the following sequence: (1) the sample was dried at a temperature of 105 °C to an air-dried state; (2) the dried sample was sieved through a 1 mm sieve; (3) the sieved sample was thoroughly mixed, and a working sample weighing at least 200 g was selected by quartering, which was then ground in a laboratory planetary ball mill to a particle size of less than 71 mm so that the yield of the 71 mm fraction was at least 95%. In the case of samples represented by pebble–sand and sand–pebble mixtures, a fraction with a particle size of 1 mm or less was used for the study (i.e., it is mainly a sand component) since the large fraction has a rather weak mechanism for the accumulation of HMes; therefore, its presence in the analyzed sample reduces the ability of any analytical method to detect the accumulation of HMes in sediments. The prepared samples were analyzed for copper (Cu), lead (Pb), zinc (Zn), cadmium (Cd), and chromium (Cr) content.

### 2.2. Chemical Analysis of Sediments

Further, the selected sediment samples were delivered to the laboratories of the University of Latvia, Faculty of Geography and Earth Sciences, and were prepared for an analysis of organic matter (OM), total nitrogen (N_tot_), total carbon (C_tot_), and phosphorus (P_2_O_5_). Chemical analysis for the detection of P_2_O_5_ concentration (mg kg^−1^) and the OM, C_tot_, and N_tot_ content (%) of the prepared samples was performed in three replicates according to the internationally used standard methods of the International Organization for Standardization (ISO) [22,23]. Total carbon and total nitrogen content (%) were determined via dry combustion (elementary analysis) by using an element analyzer, “EuroVector EA3000” (EuroVector, Redavalle, Italy). Total carbon and total nitrogen ratio (C/N) were calculated from the obtained average values. Soil phosphorus (P_2_O_5_) was determined according to the Mehlich 3 method [24]. Estimation of OM (%) was conducted according to frequently used loss of ignition method [25]. The reliability of the obtained results was assessed after chemical analysis. The laboratory results were considered acceptable when the difference between the values obtained was less than ±10%.

Concentrations of Cr, Zn, Cu, Cd, and Pb were analyzed using an inductively coupled plasma mass spectrometry ICP-MS Agilent 7500 cx manufactured by Agilent Technologies (Santa Clara, CA, USA).

The calculation of the total indicator for metal concentrations in the bottom sediment (Zc, pollution index) was carried out using Equations (1) and (2):

Kc = Ci/Cb(1)

Zc = ∑Kc − (n − 1)(2)

where Kc is concentration factor; Ci is pollutant concentration; Cb is pollutant concentration at the background point; n is the number of elements to be determined [26]. As background concentrations, the maximum permissible concentrations of the tested HMes were used [27].

### 2.3. Testing of Microbial DNA in Sediments

The metagenomic structure of microorganisms in river sediments was tested at the Latvian Biomedical Research and Study Center.

#### 2.3.1. DNA Extraction and Shotgun Sequencing

To isolate DNA from the samples, the FastDNA SPIN Kit for Soil (MP Bio-Medicals, Irvine, CA, USA) was used according to the manufacturer’s instructions. DNA samples were normalized for the shotgun metagenomic studies to an initial library input of 500 ng and afterward were sheared with a Covaris S220 Focused-Ultrasonicator (Covaris, Woburn, MA, USA) to achieve an average fragment size of 400 bp. Libraries were created using the MGIEasy Universal DNA Library Prep Set V1.0 (MGI Tech Co., Shenzhen, China), following the manufacturer’s instructions. Quality control of the libraries was evaluated using the Agilent High Sensitivity DNA kit with an Agilent 2100 Bioanalyzer (Agilent Technologies, USA) and the Qubit High Sensitivity dsDNA assay kit with a Qubit 2.0 instrument (both from Thermo Fisher Scientific, Waltham, MA, USA). The depth of the sequence was calculated to be at least 20 million reads per sample (paired-end, read length of 150 bp). To prepare DNA nanoballs (DNBs), pooled and circularized libraries were employed as templates. The PE150 flow cell was loaded based on DNBs utilizing an automated DNB loading method. Libraries were sequenced with the DNBSEQ-G400 sequencer using the DNBSEQ-G400RS High-Throughput Sequencing Set (MGI Tech Co., China), according to the established protocol.

#### 2.3.2. Shotgun Sequencing Data Analysis

Trimmomatic v.0.39 [28] was used to perform quality trimming on the collected raw paired-end reads. Sequences less than 36 nt were ignored, and the leading and trailing quality levels were set to Q30 and Q30, respectively. After quality filtering, sequences were then classified using Kraken2 [29] and RefSeq database release 98 [30], which comprises taxonomical reference data on bacterial, fungal, viral, and protozoan domains. The R-based Pavian [31] v1.0 tool was used to perform taxonomical aggregation. De novo read assembling into scaffolds was performed using the metaSPAdes [32] assembler. The generated assembly was evaluated using metaQuast [33]. The assembly database and the local alignment of reads input into the assembly were created and performed using Bowtie2. Open reading frame detection and subsequent annotation were performed using PROKKA v.1.14.6 [34] with the manually curated Swiss-Prot UniProtKB [35] database. During the annotation, predictions of rRNA, tRNA, and scaffolds below 1000 nt were excluded. Coordinates of predicted protein-coding features were used for quantification against the assembly database using HTSeq [36] and the intersection-nonempty resolution mode. Metagenomic read counts were standardized using the Transcripts Per Million method [37] with an in-house-built Python script.

#### 2.3.3. Identification of Microbial Resistance Genes

The Resistance Gene Identifier (RGI) v.5.1.1, the DIAMOND [38] alignment tool, and the Comprehensive Antibiotic Resistance Database (CARD) [39] were used to predict the resistome profile of the scaffolded metagenomes. The heat map function of the RGI was used to organize resistance genes according to gene family and resistance mechanism in order to gather results for each sample. Additionally, hierarchical clustering was carried out to group samples based on their similarity.

### 2.4. Statistical Analysis

The data presented in the tables are expressed as the mean value ± standard deviation. The differences between the treatments were assessed with Student’s *t*-test and one-way analysis of variance (ANOVA) in Microsoft Excel, Office365. Principal component analysis (PCA) of the selected sediment samples’ chemical variables (OM, P_2_O_5_, N_tot_, C_tot_, C/N ratio, Cr, Cu, Zn, Cd, P) and the abundance of resistance genes classified by drug class (DC(R)) was performed by using the PC-ORD 5.0 software. A Monte Carlo test was used to determine the significance of PCA axes. Pearson correlation coefficients (*p* < 0.05) were determined between PCA axes scores and analyzed quantitative data.

## 3. Results

### 3.1. Chemical Characterization of Sediments

Our physicochemical characterization of river sediments revealed distinct differences in OM contents, as well as biogenic elements, i.e., carbon, nitrogen, and phosphorus. The OM contents varied in a range from 0.63 to 0.64% (K9, K10) up to 6.86% (K7). The highest C_tot_ contents were detected in K6, K7, and K8, at 2.510, 2.935, and 2.384%, respectively. Samples K6, K7, and K8 were also characterized by the highest N_tot_ contents, i.e., 0.142, 0.146, and 0.082%, respectively. In turn, concentrations of P_2_O_5_ in sediments varied in a range from 8.90 mg kg^−1^ (K9) to 62.30 mg kg^−1^ (K3) (Table 2).

#### Concentration of Cr, Cu, Zn, Pb, and Cd in River Sediments and Water

The level of contamination of river sediments caused by heavy metals differed in ten sampling sites. Particularly, the highest concentration of Cr, i.e., 35.26 mg kg^−1^, was detected in Ust-Kamenogorsk, 0.8 km below the dam of the Ust-Kamenogorsk hydroelectric power station (K4). Other sites showed the presence of Cr in a range from 11.58 mg/kg in K7 to 24.26 mg kg^−1^ in K9 (Table 3). Other tested metals, i.e., Cu, Zn, Cd, and Pb, were found in the highest concentrations in K8 and K9, both within Ridder City. The highest contamination of river waters with tested metals among ten sampling sites was detected in K8 and K9, which corroborates the data on sediments. The overall degree of sediment contamination with HMes was assessed using the pollution index Zc. Taking into consideration the guidelines for soil quality in the Republic of Kazakhstan, the tested sediments ranged as follows: K8(21.6) > K4(19.6) > K9(14.5) > K1(8.3) > K3(2.1) > K10(1.98) > K5(2.0) > K6(1.14) > K2(0.45) > K7(0.43).

### 3.2. Taxonomic Profile of Sediment Samples

**Figure 2:**
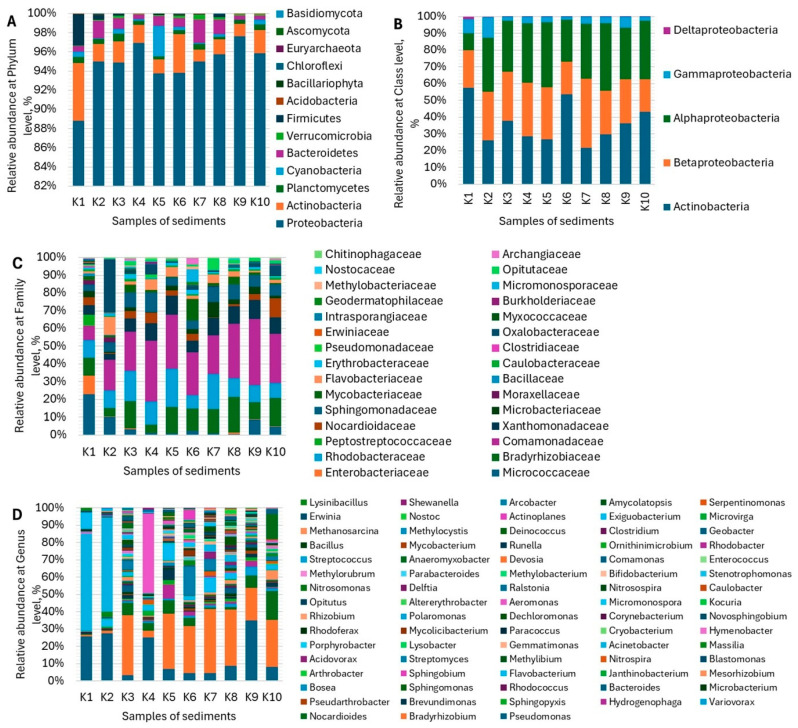
Taxonomic assignments for the sediment communities at the phylum (**A**), class (**B**), family (**C**), and genus (**D**) levels in river sediments. Only classified reads are presented with scaling to 100%. Only taxon yields ≥1% in at least one sample are shown. Abbreviations: K1—Irtysh River below the confluence; K2—Irtysh River above the water intake; K3—Ulba River within the city; K4—Irtysh River within the city below the dam; K5—Irtysh River below the wastewater discharge; K6—Krasnoyarka River in the village below the dam; K7—Krasnoyarka River within the boundaries of the village at the water station site; K8—Tikhaya River above the confluence; K9—Tikhaya River below the dam; K10—Bukhtarma reservoir.

The taxonomic profile of ten samples of river sediments is summarized in Figure 2. An examination of the metagenome showed the prevalence of Proteobacteria at the phylum level (88.84–97.61%), followed by Actinobacteria (1.21–5.98%). Other phyla appeared mostly below 1%, except Firmicutes in K1 (3.28%) and Bacteroidetes in K2, K3, K5, K7, and K8 (1.83%, 1.14%, 1.02%, 2.46%, and 1.52%, respectively). Cyanobacteria was represented in K5 by 3.17%, the most abundant phyla among the tested samples (Figure 2A). At the class level, Actinobacteria, Betaproteobacteria, and Alphaproteobacteria were the most common among the classified reads. Thus, K1, K3, K6, K9, and K10 were characterized by a dominance of Actinobacteria (36.44–57.48%), while K2, K4, K5, and K8 were characterized by Alphaproteobacteria (32.25–39.78%). Betaproteobacteria prevailed in K7 (41.17%) (Figure 2B). At the family level, the most prevalent in K1 was *Micrococcaceae* (22.51%) and *Oxalobacteraceae* in K2 (28.56%), while in the other samples (K3–K10), *Comamonadaceae* prevailed (21.14–36.72%)(Figure 2C). At the genus level, *Acinetobacter* prevailed in K1 and K2 (55.00 and 53.89%, respectively); *Bradyrhizobium* prevailed in K3, K5, K6, K7, K8, and K10 (26.14–35.56%); and *Pseudomonas* prevailed in K4 and K9 (24.89 and 34.59%, respectively) (Figure 2D). Overall, an abundance of 160 species with a yield ≥1% in at least one sample was detected. The most abundant species (a taxon yield ≥3% in at least one sample) are shown in Table 4.

A comparison of the identified functional gene families with an abundance threshold of ≥0.1% showed that the highest number of gene families were in K8 (n = 387), followed by K5 (n = 367), K6 (n = 209), and K3 (n = 161). The sample from the Bukhtarma reservoir (K10) did not contain any detectable functional gene family with a relative abundance above 0.1% (Table S1). The most diverse HMe-related gene families were in K8, where ATP-dependent zinc metalloprotease, zinc-transporting ATPase, copper-transporting ATPase, copper resistance protein A, copper-exporting P-type ATPase, and nickel–cobalt–cadmium resistance protein NccB appeared (Table S1). Sediment sample K5 contained three zinc metalloprotease FtsH types (related to the *Nostoc* sp. and two strains of the *Synechocystis* sp.) and zinc-transporting ATPase, related to *Bacillus subtilis* (Table S1). Copper-transporting P-type ATPase was identified in K2, while ATP-dependent zinc metalloprotease FtsH was identified in K3 and K6—one in each. Samples K1, K4, K7, K9, and K10 did not contain any detectable HMe-related gene families (Table S1).

#### Abundance of Antibiotic Resistance Genes

The data on antibiotic resistance genes (ARG) classified by drug class revealed an abundance of genes resistant to carbapenem, i.e., the OXA-296, OXA-363, and OXA-644 genes, in sediments K1. Also, K2 contained the beta-lactam resistance gene OXA-296. Other sediment samples did not contain resistance genes to beta-lactam antibiotics (Table S2). All tested samples were characterized by an abundance of qacJ genes resistant to disinfecting agents and antiseptics, as well as eight genes resistant to glycopeptide antibiotics (Table S2). The qacJ gene is used in small multidrug resistance (SMR) antibiotic efflux pumps, with antibiotic efflux as a resistance mechanism. In turn, genes resistant to glycopeptide antibiotics play a role in antibiotic target alteration (Table S2). Heat maps of antimicrobial resistance genes based on gene family and resistance mechanism are shown in Figure S1.

### 3.3. Principal Component Analysis (PCA) of Chemical Variables and Abundance of Resistance Genes

**Figure 3:**
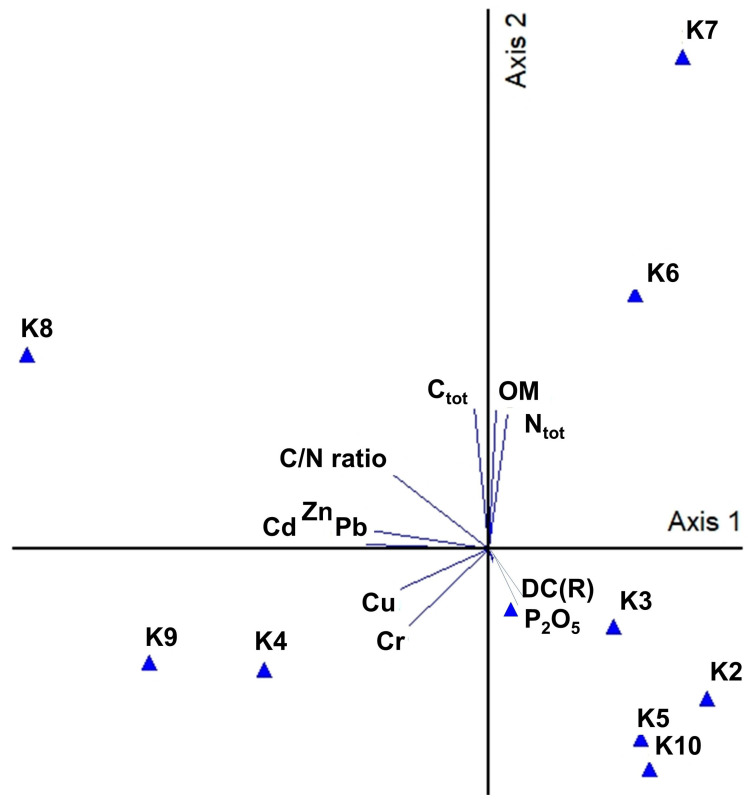
Principal component analysis (PCA) of chemical variables and abundance of resistance genes classified by drug class, i.e., DC(R). Description of chemical variables as in Table 2 and Table 3.

Ordinating the chemical variables and abundance of resistance genes from the sampling locations indicated that the first and second axes of the component analysis were statistically significant (*p* < 0.05), and together, they explain 68.94% of the total sample variation. The first axis explains 36.53% of the total variation. With the first axis, a significant (r > 0.50) positive correlation was found between Zn, Cd, P, the C/N ratio, Cu, and Cr. The second axis explains 32.41% of the total variation. With the second axis, a significant (r > 0.50) positive correlation was found between OM, Ntot, Ctot, and the C/N ratio, but with this gradient, a significant (r > 0.50) negative correlation was also found with Cr (Figure 3).

The PCA results reveal that the abundance of resistance genes classified by drug class (DC(R)) was not significantly associated with the analyzed chemical data.

## 4. Discussion

Among ten sediment samples tested in this study, K4, K8, and K9 can be classified as moderate sanitary and toxicological dangers according to the indicative scale of the Zc pollution index. The other seven sediment samples can be classified as acceptable sanitary and toxicological dangers, with a low pollution level. Sampling sites K8 and K9 are in Ridder City. In the region of East Kazakhstan, the Tikhaya River plays an important role in HMe intake, as it flows through the Ridder mining and processing complex [20]. The Tikhaya River (near the Tishinsky mine in Ridder City) continues to be the most polluted based on chemical indicators, corresponding to the “high” and “extremely high” pollution levels [40]. Sample K4—from Irtysh River sediments obtained in Ust-Kamenogorsk City, 0.8 km below the dam of the Ust-Kamenogorsk hydroelectric power station—also exhibited a comparatively high pollution level based on the tested HMes (Table 2). Importantly, in earlier studies, increased concentrations of Cr, Ni, Cu, Cd, and Pb were also detected in the soil around the tailing reservoir in the Irtysh River Basin, exceeding the soil background by 7.53, 36.08, 31.45, 35.32, and 1.76 times, respectively [41]. The sources of Cu and Zn in the soil around the tailing reservoir area are mainly the tailing reservoir area itself, while Cr, Cd, and Pb are related to transportation and mining production activities [41]. The recent analysis of HMe in surface waters of the Irtysh River analyzed the Clarkes of elements in the hydrosphere, metal spatial distribution, and changes over time at six locations. The highest values of Clarke concentrations were detected for Cd (7), Hg (2.6), and Zn (1.7), but decreased Pb and Cu contents were also found [20].

Therefore, high variability in HMe concentrations and the compositions of multi-metal mixtures in river sediments are expected to affect the microbial community structure. In our study, the metagenomic analysis revealed two dominant phyla in all tested samples, i.e., Proteobacteria and Actinobacteria (Figure 2A). These results corroborate other studies on HMe-polluted rivers that were conducted in China [42,43] and Taiwan [44]. Culturable bacteria belonging to these phyla and resistant to HMe have been obtained from river sediments in the Teesta River in the Eastern Himalayas [45] and Poland [46]. In our study, Proteobacteria were represented mostly by Alphaproteobacteria and Betaproteobacteria at the class level (Figure 2B). Among Betaproteobacteria, 15 taxa of *Pseudomonas* spp. were identified. *Pseudomonas* is known to be involved in various HMe transportation mechanisms, particularly for Ni and Zn, suggesting that these bacteria could be used for heavy metal remediation [47]. Also, *Rhodobacteraceae* has been shown to be predominant in HMe-contaminated river ecosystems in the residential and mining area [48]. Our data also demonstrated a high relative abundance of this taxon in all samples, ranging from 7.17% in K6 up to 21.11% in K5 (Figure 2C). Liu et al. reported that *Sphingomonadaceae* and Cyanobacteria play an important role in the bacterial communities of polluted rivers [49]. As shown in Figure 2C, *Sphingomonadaceae* appeared in relatively high concentrations in all 10 samples, i.e., from 3.45% to 10.71%, the highest in K4. In turn, the highest concentration of Cyanobacteria (3.17%) was detected in K5, while in other samples, this taxon varied in a range from 0.03% to 0.55% (Figure 2A). As reported by Zhao et al. [50], in river sediments severely polluted with 1704 mg/kg of Zn and 1.92 mg/kg of As, *Acinetobacter johnsonii*, *Clostridium cellulovorans*, and *Trichococcus pasteurii* were the dominant bacteria. Our data showed the dominance of *Acinetobacter johnsonii* in K1 and K2 (i.e., 14.07% and 7.00%, respectively), where the concentration of Zn was not the highest among ten samples (Table 2 and Table 3). Interestingly, in total, 12 *Acinetobacter* species were identified in samples K1 and K2 with a relative abundance ranging from 1% up to 15.74% (*Acinetobacter lwoffii* in K1). At the genus level, the abundance of *Acinetobacter* in K1 and K2 reached 55.00% and 53.89%, respectively (Figure 2D). Not only does Zn stimulate the proliferation of *Acinetobacter* spp. in contaminated river sediments, but other environmental factors probably do as well. The abundance of *Clostridium* spp. was negligible, and *Trichococcus* spp. were not detected in the tested samples. Instead, the *Aeromonas* genus was dominant in K4 with a relative abundance of 46.02% (Figure 2D). In a study on HMe-resistant *Aeromonas* spp. isolated from the Ba River in Northwest China, *Aeromonas veronii* was shown to be more adaptable to contaminated waters [51]. Our data showed the presence of *A. veronii* only in K4 (9.33%) (Table 4). Recently, Fakhar et al. [52] reviewed the resistance mechanisms of *Aeromonas*, *Bacillus*, and *Pseudomonas* in soil regarding HMe remediation.

Our analysis of ARGs in K1 and K2 revealed an abundance of carbapenem-resistant genes related to OXA-type β-lactamases (Class D carbapenemases), which act as antibiotic inactivators and are known to be of clinical relevance (Table S2). β-lactamase inhibitors (e.g., amoxicillin–clavulanic acid, etc.) are effective in clinical therapy for class A β-lactamases but do not inhibit class D carbapenemases [53]. Regarding the prevalence of the qacJ resistance gene in all tested samples, its resistance mechanism is based on antibiotic efflux (Table S2). [54] identified this plasmid-borne gene as encoding resistance to quaternary ammonium compounds in *Staphylococcus aureus* and showed its horizontal transfer [54]. Nevertheless, the most HMe-contaminated sediments (e.g., K4, K8, K9) were characterized by a considerably lower number of ARGs compared with K1 (Table S2). Apparently, sampling site K1—situated in Ust-Kamenogorsk in the Irtysh River, 2 km below the confluence with the Ulba River—could be heavily influenced by other anthropogenic factors, not by HMe contamination. In turn, the Bukhtarma reservoir (K10) could be considered to have minimum anthropogenic pressure among the tested samples.

Data from the literature show that, among major factors influencing the microbial community structure in HMe-contaminated freshwater sediments, a specific composition of HMe and the physicochemical properties of water and sediments significantly affect the shape of the microbial community’s structure [43,48,55,56]. In a study of soil, Cr pollution antagonistically affected both some nutrient elements (organic matter, P, K, Ca, Mg) and some heavy metals (Fe, Cu, Zn, Mn) [57,58]. The absorption capacity, the grain size composition, the acidity, the number of exchangeable cations, and other parameters influence the mobility and accumulation of HMes in river sediments [26]. The chemical characteristics of sediments tested in the present study indicated that ten sampling sites considerably differed in OM, C_tot_, and N_tot_ contents and concentrations of P_2_O_5_ (Table 2). This could be explained by differences in the characteristics of riverbed-formation-influencing processes and factors in sediment accumulation (water flow specifics, slope gradient, etc.). Sediments sampled in Glubokoye District (K6), Predgornoie (K7), and Ridder City (K8) had comparatively high OM, C_tot_, and N_tot_ content, while increased concentrations of P_2_O_5_ were detected in Ustj-Kamenogorsk (K1–K4), as well as in Glubokoye District 60m below the hydraulic dam (K6), Ridder city 0.1 km above the technological road (K8), and the Bukhtarma reservoir (K10). The obtained results indicate the anthropological contamination of these sites. Therefore, the fate of contamination with HMe, e.g., metal sorption capacity, mobility, eco-toxicity, and remediation processes, is expected to be considerably different. These differences, as well as seasonality and river hydrological cycles, can also influence the microbial community’s structure in sediments. Specifically, drainage, precipitation, and flow rate significantly correlate; therefore, these processes possibly influence the distribution of HMes [59].

Regarding the characteristics of the microbiome, it should be noted that a relatively low yield of classified reads in the tested samples was obtained (Table A1). The unclassified parts of microbiological matter are very valuable for research; however, a lack of a unified system for their identification remains the main obstacle in metagenome annotation for environmental samples, guts, and others [60]. In this regard, it is still necessary to increase the completeness of these databases. In studies of the gut microbiome, the application of different classification methods (e.g., Kraken2 and Metaphlan2) has provided quite divergent results [61]. To reduce the proportion of unclassified reads, the modification of reference index databases has been proposed, as compared with using a default NCBI RefSeq database [62]. Thus, it is believed that this limitation will be solved in the future.

## 5. Conclusions

By summarizing the results obtained in this study, the following conclusions can be made:

- Ten samples of riverbed sediments taken from different sites in the East Kazakhstan region considerably contrasted in their physicochemical characteristics and contamination with HMes. The highest HMe concentrations were detected in Ridder City (K8 and K9) and Ust-Kamenogorsk City, 0.8 km below the dam of the hydroelectric power station (K4). These samples were also characterized by the highest total number of identified functional gene families, including those related to Zn, Cu, Ni, Co, and Cd.
- The prevalence of Proteobacteria and Actinobacteria at the phylum level was shown in all ten samples examined in this study. At lower taxonomic ranks, the tested sediments differed in dominant bacteria. The data on the prevalence of bacteria taxa in river sediments at historically contaminated sites represent an important tool for the further investigation of microbial multi-resistance toward HMe, as well as other substances (e.g., antibiotics).
- The most HMe-contaminated sediments (i.e., K4, K8, K9) were characterized by a considerably lower number of ARGs compared with K1. Apparently, sampling site K1—situated in Ust-Kamenogorsk in Irtysh River, 2 km below the confluence with the Ulba River—may be heavily influenced by other anthropogenic factors, not by HMe contamination.
- A lack of data on the concentration of other pollutants (e.g., other metals, hydrocarbons, S- and F-containing molecules, etc.), seasonal variables, the spatial distribution of contamination, and other factors means that we might be underestimating the selective pressure of living surrounding on the microbial community structure in riverbed sediments. Further study is needed for a deeper understanding of the interplay between mining activities, urbanization, environmental changes, and the response of microorganisms as the main inhabitants among benthic organisms. New knowledge in this field will provide essential insights into sustainable river management in the context of intensive mining and metallurgy activities.

## Figures and Tables

**Table 1:** Location and description of sampling sites.

Sample No.	Geographical Coordinates of Sampling Sites	Name of the Location	Description of Sampling Sites
K1	49°57′30.6″ N 82°34′16.7″ E	Ust-Kamenogorsk, 070000, Kazakhstan	Irtysh River 2 km below the confluence with the Ulba River (Samal district)
K2	49°55′36.3″ N 82°40′10.6″ E		Irtysh River, 3 km above the Pionersky water intake (Ablaketka District)
K3	49°57′15.0″ N 82°36′45.0″ E		Ulba River, Ust-Kamenogorsk, within the city; 1 km above the mouth of the Ulba River; 0.36 km below the Ulbinsky bridge; right bank
K4	49°53′29.0″ N 82°42′12.0″ E		Irtysh River, Ust-Kamenogorsk, within the city; 0.8 km below the dam of the Ust-Kamenogorsk hydroelectric power station; at the water post site; right bank
K5	49°55′37.0″ N 82°40′10.0″ E		Irtysh River, 0.5 km below the wastewater discharge of the capacitor plant, 0.5 km above the railway bridge, right bank
K6	50°15′16.0″ N 82°21′47.0″ E	Glubokoye District, 070000, Kazakhstan	Krasnoyarka River, within Altai Village; 60 m below the hydraulic structure (dam); 24 km above the mouth of the Krasnoyarka River; right bank
K7	50°14′50.0″ N 82°14′39.0″ E	Predgornoje, 070000, Kazakhstan	Krasnoyarka River, within the boundaries of the village of Predgornoye; 3.5 km above the mouth; at the water station site; right bank
K8	50°21′26.0″ N 83°28′53.0″ E	Ridder, 070000, Kazakhstan	Tikhaya River, 0.1 km above the technological road bridge; 0.17 km above the confluence of the Bezymianny stream; left bank
K9	50°19′17.0″ N 83°25′52.73″ E		Tikhaya River, 0.23 km below the hydraulic structure (dam); 8 km above the mouth of the Tikhaya River; left bank
K10	49°37′10.0″ N 83°26′25.4″ E	Bukhtarma Reservoir, 070000, Kazakhstan	Bukhtarma Reservoir

**Table 2:** Organic matter (OM), nitrogen (N_tot_), and carbon (C_tot_) content (%) and concentration (mg kg^−1^) of phosphorus (P_2_O_5_) in the tested river sediment samples.

Sample	OM, %	P_2_O_5_, mg kg^−1^	N_tot_, %	C_tot_, %	C/N Ratio
K1	1.52	45.70 ± 0.71	0.029 ± 0.003	0.696 ± 0.177	23.98
K2	1.02	49.25 ± 1.48	0.023 ± 0.012	0.254 ± 0.179	11.27
K3	2.27	62.30 ± 1.27	0.064 ± 0.012	0.703 ± 0.045	11.07
K4	2.07	45.60 ±5.94	0.036 ± 0.022	0.730 ± 0.224	20.55
K5	0.70	25.35 ± 1.34	0.011 ± 0.002	0.098 ± 0.023	9.33
K6	3.92	38.30 ± 1.41	0.142 ± 0.002	2.510 ± 0.260	17.74
K7	6.85	13.55 ± 0.49	0.146 ± 0.001	2.935 ± 0.005	20.17
K8	3.76	40.55 ± 0.49	0.082 ± 0.001	2.384 ± 0.547	29.25
K9 *	0.63	8.90 ± 0.00	0.019 ± 0.003	0.377 ± 0.042	20.20
K10 *	0.64	37.10 ± 0.00	0.022 ± 0.006	0.123 ± 0.002	5.51

* The samples were sieved through a 0.025 cm sieve to remove the largest mineral particles.

**Table 3:** Concentration of heavy metals in river sediments.

Sample	Cr	Cu	Zn	Cd	Pb
	mg kg^−1^	mg kg^−1^	mg kg^−1^	mg kg^−1^	mg kg^−1^
K1	23.41	8.81	33.87	3.23	5.56
K2	16.67	14.87	12.19	0.25	6.01
K3	20.59	19.56	22.58	0.41	8.66
K4	35.26	16.10	42.04	7.47	13.90
K5	21.72	15.92	18.35	0.46	5.69
K6	18.27	16.63	16.83	0.34	5.61
K7	11.58	6.61	27.67	0.46	5.75
K8	23.28	25.11	67.35	8.53	32.00
K9	24.26	23.73	53.04	5.12	36.70
K10	22.67	17.03	24.70	0.24	4.08

**Table 4:** The microbial community structures at the species level in river sediments. Only taxon yields ≥3% in at least one sample are shown (marked in grey color). A description of the samples is summarized in Figure 1 and Table 1.

Species	K1	K2	K3	K4	K5	K6	K7	K8	K9	K10
*Acidovorax sp. JMULE5*	0.04	0.02	0.23	1.66	1.23	0.96	10.08	0.46	2.00	0.11
*Acinetobacter baumannii*	0.14	3.06	0.01	0.00	0.01	0.00	0.03	0.09	0.00	0.02
*Acinetobacter johnsonii*	14.07	6.99	0.02	0.03	0.01	0.02	0.06	0.28	0.01	0.07
*Acinetobacter lwoffii*	15.74	1.63	0.02	0.03	0.02	0.07	0.03	0.04	0.02	0.04
*Acinetobacter sp. ACNIH2*	3.50	2.61	0.01	0.01	0.03	0.02	0.02	0.02	0.00	0.01
*Actinoplanes derwentensis*	0.00	0.00	0.15	0.00	0.00	0.15	0.03	14.06	0.00	0.00
*Aeromonas salmonicida*	0.02	0.02	0.02	6.88	0.24	0.70	0.03	0.06	0.00	0.08
*Aeromonas sp. CU5*	0.00	0.12	0.07	34.73	0.17	0.09	0.01	0.01	0.00	0.43
*Aeromonas veronii*	0.01	0.05	0.04	9.33	0.08	0.11	0.02	0.10	0.00	0.17
*Archangium gephyra*	0.52	0.00	6.00	0.00	0.66	5.70	0.01	0.01	0.02	3.87
*Arthrobacter sp. FB24*	0.44	2.21	0.56	0.04	0.15	0.01	0.02	0.04	1.07	6.02
*Bosea sp. RAC05*	0.02	1.79	9.59	0.02	0.69	0.55	0.13	1.52	1.09	0.62
*Burkholderiales bacterium JOSHI_001*	0.03	0.09	0.63	2.37	2.60	0.69	1.04	0.70	0.95	6.40
*Buttiauxella sp. 3AFRM03*	3.01	0.09	0.00	0.00	0.00	0.00	0.00	0.01	0.00	0.02
*Caulobacteraceae bacterium OTSz_A_272*	0.00	0.07	0.39	0.72	0.39	0.20	0.18	0.14	0.61	0.86
*Exiguobacterium mexicanum*	3.96	0.20	0.06	0.00	0.02	0.64	0.02	0.00	0.00	0.02
*Flavobacterium sp. Sr18*	0.44	7.67	0.05	0.30	0.22	0.04	0.54	0.29	0.11	2.37
*Gemmatimonas phototrophica*	0.01	1.23	8.31	0.03	0.47	1.29	2.72	2.30	0.80	1.05
*Hydrogenophaga sp. RAC07*	0.01	0.03	0.44	0.31	7.48	0.14	1.35	1.50	5.20	0.78
*Microbacteriaceae bacterium WY83*	0.21	0.04	0.24	0.00	5.00	0.03	1.45	0.22	4.27	0.01
*Microcoleus sp. PCC 7113*	0.00	0.00	2.41	0.00	0.00	3.81	0.00	0.00	0.04	0.01
*Micromonospora zamorensis*	0.02	0.13	0.05	0.02	0.01	3.44	0.29	0.06	0.05	0.01
*Microvirga ossetica*	0.12	0.06	1.33	0.16	0.10	17.81	0.87	0.19	0.06	0.02
*Modestobacter marinus*	0.01	0.01	0.48	0.02	0.01	7.83	0.05	0.07	0.50	0.09
*Mycolicibacterium arabiense*	0.01	0.01	0.13	0.00	0.03	4.91	0.09	0.05	0.02	0.01
*Nitrosomonas sp. Is79A3*	0.01	0.09	0.81	0.18	4.72	0.41	0.64	3.24	0.55	2.50
*Nitrospira defluvii*	0.07	0.06	0.75	0.13	1.46	1.13	10.10	7.20	1.93	0.38
*Novosphingobium ginsenosidimutans*	0.09	4.50	6.24	0.15	1.44	0.43	2.44	1.06	0.13	2.15
*Opitutus sp. GAS368*	0.00	0.25	2.11	0.46	0.91	1.03	11.79	1.65	3.39	3.06
*Pantoea agglomerans*	10.81	0.07	0.02	0.02	0.02	0.25	0.06	0.04	0.00	0.02
*Planctomycetes bacterium ETA_A1*	0.14	0.47	4.08	0.34	1.77	3.15	2.87	1.31	1.29	8.19
*Planctomycetes bacterium I41*	0.07	1.66	9.53	0.10	0.63	0.34	0.17	0.31	1.59	7.13
*Pseudomonas entomophila*	0.01	3.02	0.05	0.00	0.00	0.00	0.05	0.01	0.02	0.01
*Pseudomonas putida*	0.58	4.82	0.07	0.03	0.04	0.04	0.05	0.13	1.50	0.05
*Pseudomonas silesiensis*	12.19	11.56	0.02	0.34	0.23	0.06	0.20	0.02	3.76	0.07
*Pseudomonas sp. UW4*	0.06	0.25	0.01	0.52	0.00	0.02	0.05	0.01	8.92	0.01
*Pseudomonas umsongensis*	0.94	0.77	0.01	0.03	0.13	0.01	0.02	0.03	7.04	0.01
*Rhodoferax sp. BAB1*	0.01	0.92	0.40	0.02	5.79	2.93	0.51	0.40	2.45	1.78
*Serpentinomonas mccroryi*	0.00	0.01	0.03	0.01	0.02	0.02	0.09	3.99	0.01	0.02
*Sphingosinicella sp. BN140058*	0.00	0.00	0.02	0.00	0.01	4.10	0.00	0.00	0.01	0.00
*Sulfuricaulis limicola*	0.01	0.19	0.19	0.04	0.20	0.03	0.52	0.09	0.02	5.12
*Tabrizicola piscis*	0.53	0.53	1.95	0.15	35.25	0.92	1.51	0.89	0.82	1.09
*Undibacterium parvum*	0.00	0.04	0.04	19.35	0.17	0.00	0.02	0.21	0.05	0.13

## Data Availability

The data are available upon request.

## Supplementary Materials

The following supporting information can be downloaded at https://www.mdpi.com/article/10.3390/microorganisms12020246/s1: Table S1: Relative abundance of functional gene families in river sediments. Table S2: Abundance of antibiotic resistance genes in river sediments. Figure S1: The differences in specific ARG family presence in the river sediments.

## Appendix A

**Table A1:** The number of filtered reads that were subjected to classification and the proportion of classified reads in DNA from river sediments.

Name	Number of Raw Reads	Classified Reads	Chordate Reads, %	Bacterial Reads, %	Viral Reads, %	Fungal Reads, %	Protozoan Reads, %
K1	34492563	13.50%	1.85 × 10^−5^	13.30%	2.71 × 10^−5^	2.01 × 10^−4^	2.26 × 10^−7^
K2	26690851	6.07%	5.21 × 10^−6^	5.97%	5.73 × 10^−6^	1.39 × 10^−5^	8.62 × 10^−7^
K3	27166997	4.09%	1.69 × 10^−5^	3.99%	1.33 × 10^−6^	1.17 × 10^−5^	4.79 × 10^−7^
K4	41615524	3.79%	1.56 × 10^−6^	3.69%	9.61 × 10^−7^	4.81 × 10^−6^	5.77 × 10^−7^
K5	14414917	4.21%	2.15 × 10^−6^	4.10%	5.55 × 10^−7^	6.04 × 10^−6^	2.77 × 10^−7^
K6	28902876	5.25%	3.81 × 10^−6^	5.12%	1.28 × 10^−6^	2.96 × 10^−5^	9.34 × 10^−7^
K7	39748441	4.00%	7.45 × 10^−6^	3.89%	3.47 × 10^−6^	1.73 × 10^−5^	7.80 × 10^−7^
K8	23419659	4.53%	3.83 × 10^−5^	4.42%	3.33 × 10^−6^	1.67 × 10^−5^	7.69 × 10^−7^
K9	26108461	6.09%	1.40 × 10^−5^	5.98%	1.11 × 10^−6^	1.18 × 10^−5^	4.98 × 10^−7^
K10	41973872	4.53%	4.19 × 10^−6^	4.41%	1.83 × 10^−6^	1.71 × 10^−5^	7.86 × 10^−7^
