# Supplementary material for: Impact of Anthropogenic Activities on Microbial Community Structure in Riverbed Sediments of East Kazakhstan
Source: Microorganisms. 2024 Jan 24;12(2):246. doi: 10.3390/microorganisms12020246 (PMC10893015; doi:10.3390/microorganisms12020246)

Figure S1. The differences in specific ARG family presence in the river sediments. Yellow colour—100% sequence match, bluish colour—>90% match, dark purple—not identified. Genes with asterisks (*) appear multiple times because they belong to more than one AMR gene family. The heat map of differences in specific resistance genes in the river sediments visualized according to gene family (A), and resistance mechanism (B). Abbreviations: K1 – Irtysh River below the confluence; K2 – Irtysh River above the water intake; K3 – Ulba River within the city; K4 – Irtysh River within the city below the dam; K5 – Irtysh River below the wastewater dicharge; K6 – Krasnoyarka River in the village below the dam; K7 – Krasnoyarka River within the boundaries of the village at the water station site; K8 – Tikhaya River above the confluence; K9 – Tikhaya River below the dam; K10 – Bukhtarma reservoir.

A. Heatmap of gene family.

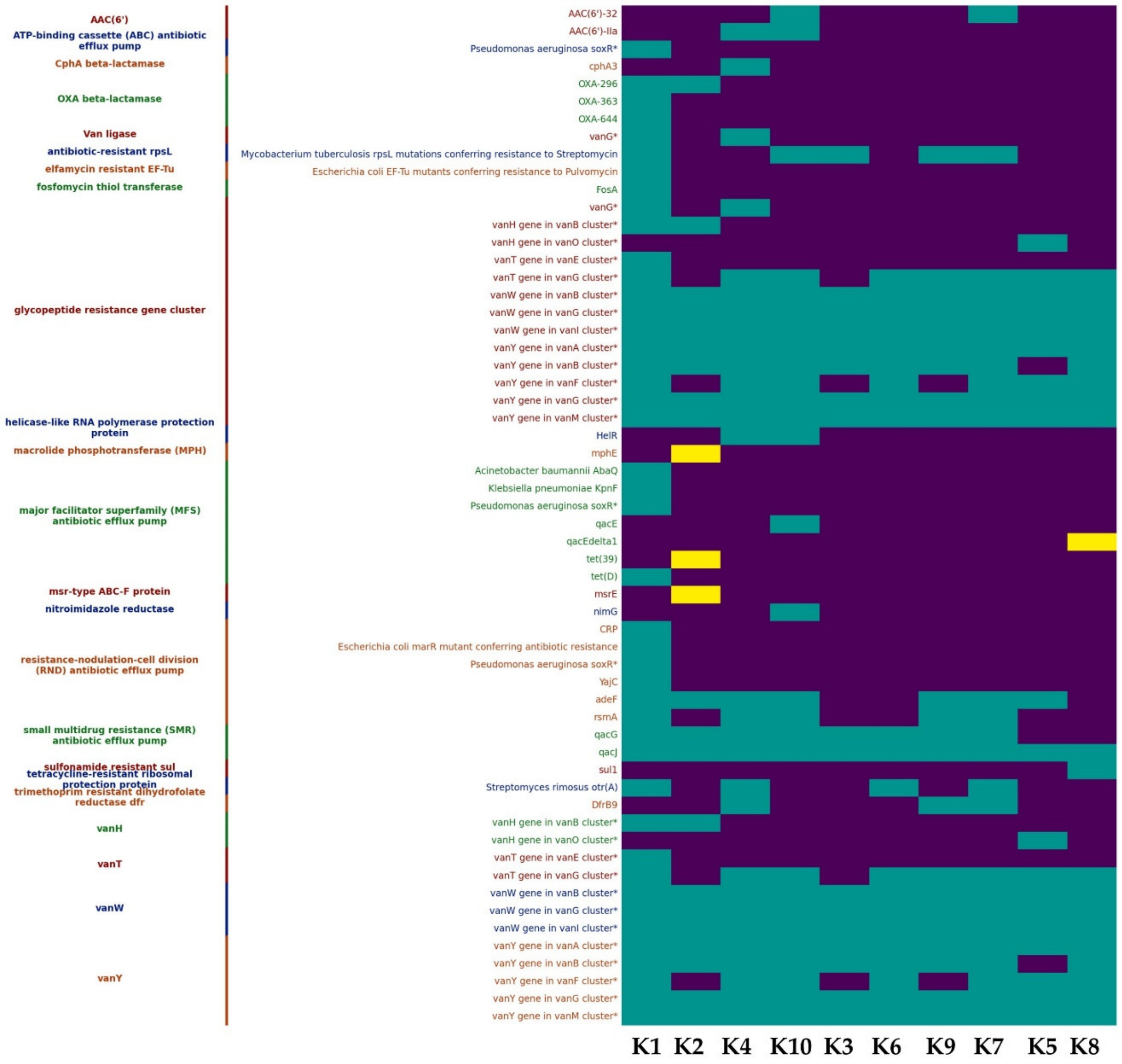

B. Heatmap of resistance mechanisms.

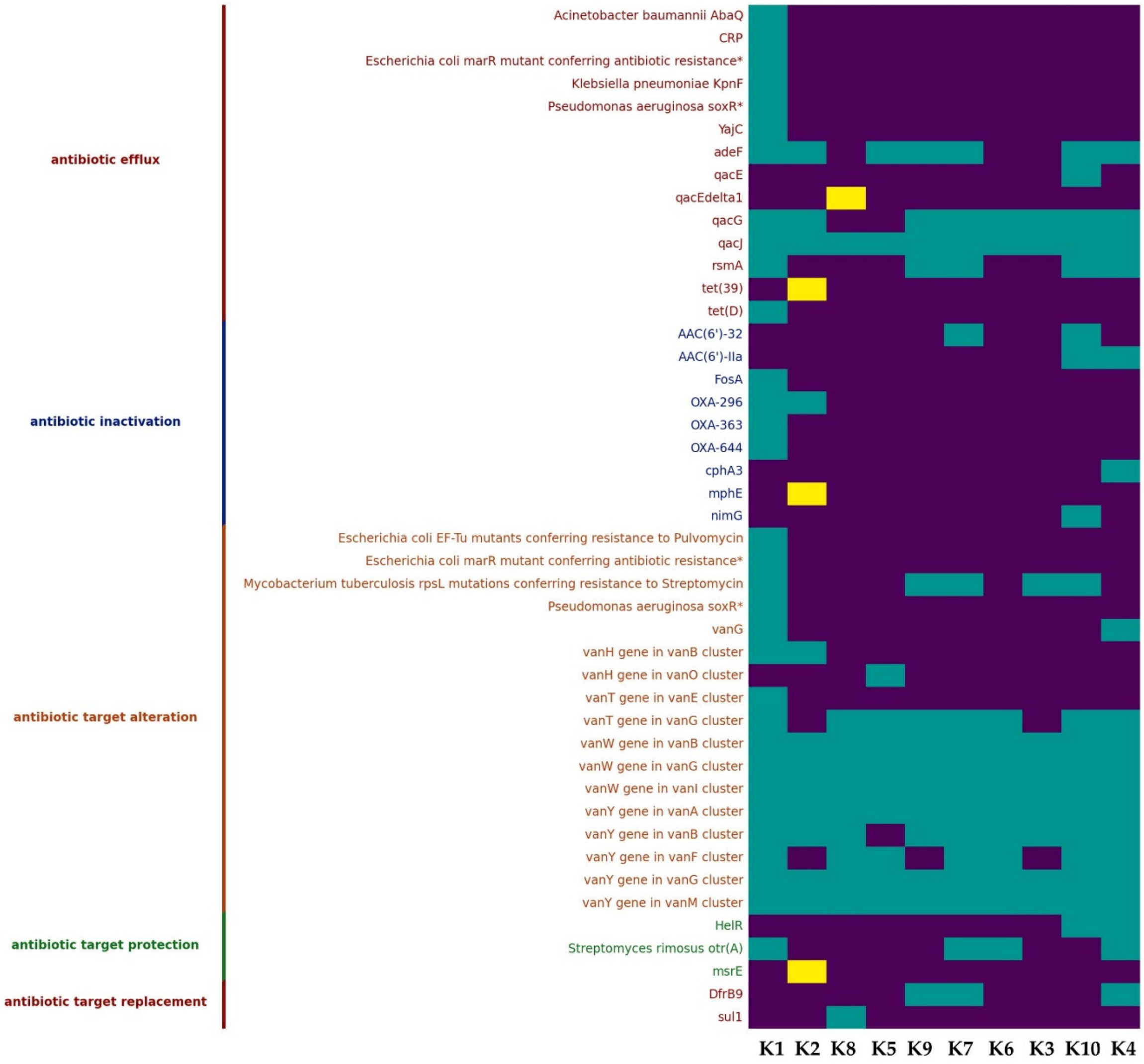

Supplement: Supplementary file 1 [file microorganisms-12-00246-s001.zip › Figure S1.pdf]
